# Evaluation of the Path-Tracking Accuracy of a Three-Wheeled Omnidirectional Mobile Robot Designed as a Personal Assistant

**DOI:** 10.3390/s21217216

**Published:** 2021-10-29

**Authors:** Jordi Palacín, Elena Rubies, Eduard Clotet, David Martínez

**Affiliations:** Robotics Laboratory, Universitat de Lleida, Jaume II, 69, 25001 Lleida, Spain; helenarubies@gmail.com (E.R.); eduard.clotet@udl.cat (E.C.); david.martinez@udl.cat (D.M.)

**Keywords:** omnidirectional mobile robot, omnidirectional wheel, path tracking, kinematic model, inverse kinematic model

## Abstract

This paper presents the empirical evaluation of the path-tracking accuracy of a three-wheeled omnidirectional mobile robot that is able to move in any direction while simultaneously changing its orientation. The mobile robot assessed in this paper includes a precise onboard LIDAR for obstacle avoidance, self-location and map creation, path-planning and path-tracking. This mobile robot has been used to develop several assistive services, but the accuracy of its path-tracking system has not been specifically evaluated until now. To this end, this paper describes the kinematics and path-planning procedure implemented in the mobile robot and empirically evaluates the accuracy of its path-tracking system that corrects the trajectory. In this paper, the information gathered by the LIDAR is registered to obtain the ground truth trajectory of the mobile robot in order to estimate the path-tracking accuracy of each experiment conducted. Circular and eight-shaped trajectories were assessed with different translational velocities. In general, the accuracy obtained in circular trajectories is within a short range, but the accuracy obtained in eight-shaped trajectories worsens as the velocity increases. In the case of the mobile robot moving at its nominal translational velocity, 0.3 m/s, the root mean square (RMS) displacement error was 0.032 m for the circular trajectory and 0.039 m for the eight-shaped trajectory; the absolute maximum displacement errors were 0.077 m and 0.088 m, with RMS errors in the angular orientation of 6.27° and 7.76°, respectively. Moreover, the external visual perception generated by these error levels is that the trajectory of the mobile robot is smooth, with a constant velocity and without perceiving trajectory corrections.

## 1. Introduction

The popularity of vehicles using an omnidirectional motion system in the field of robotics is on the rise [1,2,3]. An omnidirectional motion is one of the principal requirements for mobile robots designed to operate in complex and unstructured environments in order to develop services such as workshop assistance [4], domestic [5] or home assistance [6] and health-care assistance [7]. The main benefit of an omnidirectional motion system is that it provides three degrees of freedom (DOFs) in a ground plane, allowing displacements in any direction while changing its orientation. This omnidirectional mobility is usually achieved with the use of three or four omnidirectional wheels.

The main drawback of using a mobile robot with omnidirectional wheels is the evaluation of the odometry because the odometry is highly influenced by the practical implementation details such as the mechanical performances of the omnidirectional wheels, the accuracy of the direct and inverse kinematic models, the electrical performances of the motors used to drive the wheels, the rotary encoders used to estimate the angular rotational velocity of the wheels [8] and the tuning of the motor controllers. For example, Tsai et al. [9] and Tri et al. [10] proposed the implementation of omnidirectional mobile robots using three double-line omnidirectional wheels (also known as double parallel wheels), easy to manufacture but with the drawback of having two radial distances relative to the center of the mobile robot depending on the inner or outer parallel wheel in effective contact with the ground. In this case, this duality originates the existence of eight different kinematic models and additional mechanical constraints during a displacement. Nevertheless, the model complexity originated by the use of double-line parallel wheels and other similar wheels is usually addressed by computing an average radial distance for the wheel. For example, Lin et al. [11] addressed the trajectory errors originated by a mobile robot using double-line omnidirectional wheels by implementing a calibration procedure. In a similar direction, Maddahi et al. [12] proposed a calibration procedure applied to a lightweight mobile robot using three simple single-line omnidirectional wheels (also known as omnidirectional wheels with multiple passive rollers). In this case, this procedure assumes that the motors of the mobile robot are able to reach instantaneously the target angular rotational speed. The importance of the control of the motors used to drive the wheels of an omnidirectional mobile robot was specifically highlighted and analyzed by Li et al. [13] and by Tri et al. [10] in the case of using several omnidirectional configurations. Finally, there are many alternatives that can be used to control the trajectory of an omnidirectional mobile robot such as the use of PID controllers [14], the use of a model predictive control strategy (MPCS) [15] or the use of the anisotropic characteristics of the mobile robot [16]. Alternatively, the motion control implemented in the mobile robot assessed in this paper is based on the deterministic computation of the motion command required to reach the next discrete position and orientation.

### New Contribution

This paper is inspired by the work of Li et al. [13] that presented the simulation of some trajectories performed by a three-wheeled omnidirectional mobile robot and also analyzed the limitations found in a real implementation of these trajectories. In this direction, the new contribution of this paper is the experimental evaluation of the path-tracking accuracy of the omnidirectional mobile robot designed at the University of Lleida [17] to operate as an assistant personal robot (APR). This mobile robot has been used to develop several assistive services, but the accuracy of its path-tracking system has not been specifically evaluated until now.

Previously, Moreno et al. [18] analyzed in 2016 the basic motion performance of the first mobile robot prototype designed as an APR (named APR-01 [17]). This first prototype was designed for robotic services requiring only remote manual tele-control and did not have path-planning and path-following capabilities as it was not planned to move autonomously. The experimentation with the APR-01 fostered the development of a second improved prototype (named APR-02) designed to fully operate autonomously in unstructured environments. The main improvement of the APR-02 prototype was the development of a path-planning and path-following procedure that has been widely used to develop complex operations such as gas leakage detection [19].

The new contribution of this paper is, then, the empirical evaluation of the path-tracking accuracy of the omnidirectional mobile robot APR-02 in terms of the maximum absolute error and the root mean square (RMS) error of the planned position of the mobile robot and of the planned angular orientation of the mobile robot, which is not usually evaluated. The particularity of the APR-02 is the application of a path-following procedure based on the information gathered by its precise onboard LIDAR. This information was also registered to obtain the ground truth trajectory of the mobile robot in order to estimate the path-tracking accuracy of each experiment conducted. The path-planning procedure implemented was based on the deterministic computation of the motion command required to reach the next planned mobile robot position and orientation. Finally, the accuracy of the resulting path-tracking performances was evaluated following standard target trajectories and moving at different translational velocities.

## 2. The Mobile Robot APR

**Figure 1:**
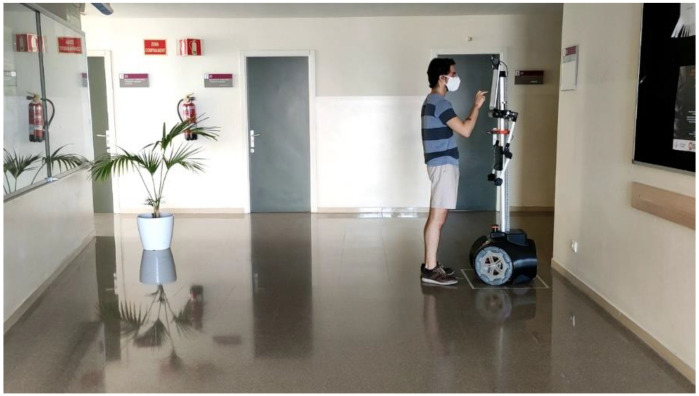
Image of one of the authors of the paper preparing the mobile robot APR-02 for an autonomous exploration under COVID-19 restrictions.

Figure 1 shows an image of the mobile robot APR-02 ready to initiate an autonomous exploration. The APR-02 is an indoor omnidirectional mobile robot prototype developed by the Robotics Laboratory of the University of Lleida [17]. The mobile robot APR-02 is a tall and thin (1700 × 55 mm) indoor humanoid mobile robot with a touch screen monitor as a head, two simple thin arms with four degrees of freedom (DOFs) and a decorative hand. The mobile robot has a weight of 31 kg and a hexagonal base with a diameter of 540 mm, with structural pieces made of aluminum and non-structural pieces made of PLA and ABS using 3D printing. The omnidirectional motion system is composed of three omnidirectional wheels optimized to operate on flat floors [18]. This mobile robot has been designed to fulfill the requirement to operate in indoor collaborative domestic environments [20] and also in indoor industrial environments [21]. The mobile robot APR-02 has been optimized during several years to develop applications requiring autonomous navigation [19,22] based on the information gathered by an onboard precise LIDAR (UTM-30LN with 270°, 1.081 scan points, 40 Hz scan rate, 30 m range and individual scan precision from ±10 to ±50 mm) and SLAM [23]. At this moment, the next planned evolution of the APR prototypes is the development of outdoor applications based on the use of all-terrain omnidirectional wheels [24] and push-broom LIDARs [25] to improve the detection of obstacles in outdoor environments.

The omnidirectional motion system used in the family of APR mobile robots was described in Moreno et al. [18]. This motion system is based on three optimal omnidirectional wheels shifted 120° from each other (also known as a kiwi drive): the distance between the center of the robot and each wheel $\left(R_{a},R_{b},R_{c}\right)$ is 195 mm, and the radius of the wheels $\left(r_{a},r_{b},r_{c}\right)$ is 148 mm. The design of the omnidirectional wheels is considered optimal because the transversal rotating rollers have a minimized gap distance. The omnidirectional wheels are driven by geared brushed DC motors with a low-cost magnetic rotary encoder attached. The estimation of the angular rotational speed of the DC motors is performed by measuring and processing the pulse length of the digital signal provided by the encoder [8]. The omnidirectional motion system can move the robot up to 1.0 m/s in any direction, although 0.3 m/s is the nominal translational velocity used in most of its applications [19].

## 3. Kinematics of the Omnidirectional Mobile Robot APR

**Figure 2:**
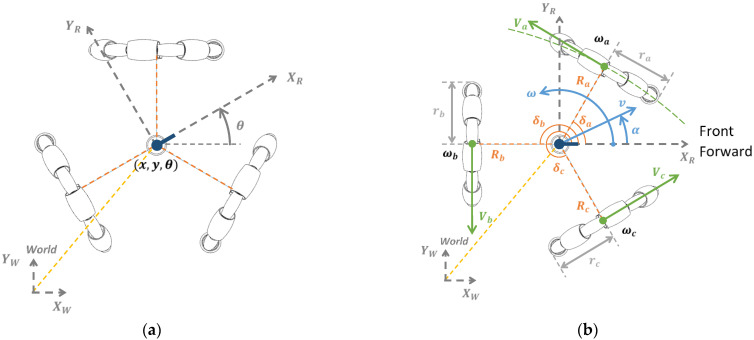
(**a**) Representation of the location of the center of the mobile robot (x,y) and absolute angular orientation (θ) of the omnidirectional mobile robot relative to the world reference frame $\left(X_{W},Y_{W}\right)$ . The reference frame $\left(X_{R},Y_{R}\right)$ is the mobile frame of the mobile robot, where the axis $X_{R}$ depicts the front of the mobile robot. (**b**) Representation of the parameters of a motion vector (v,α,ω) . $\left(R_{a},R_{b},R_{c}\right)$ are the radial distances of each omnidirectional wheel relative to the center of the mobile robot, $\left(\delta_{a},\delta_{b},\delta_{c}\right)$ are the angular orientations of the wheels relative to the mobile robot reference frame $\left(X_{R},Y_{R}\right)$ , and $\left(\omega_{a},\omega_{b},\omega_{c}\right)$ are the representation of the angular rotational speed of the wheels.

Figure 2a,b illustrate the reference frames and parameters of the omnidirectional motion system of the mobile robot APR used to define the kinematics of the mobile robot. Figure 2a shows the position and orientation of the center of the omnidirectional mobile robot in the world reference frame $\left(X_{W},Y_{W}\right)$ , referenced as (x,y,θ) . The value of (x,y) represents the translation of the mobile robot reference frame $\left(X_{R},Y_{R}\right)$ relative to the fixed world reference frame $\left(X_{W},Y_{W}\right)$ , whereas θ is the angular rotation of the reference frame of the mobile robot $\left(X_{R},Y_{R}\right)$ relative to the fixed world reference frame $\left(X_{W},Y_{W}\right)$ . Figure 2b represents the structural parameters $\left(R_{a},R_{b},R_{c}\right)$ , which are the radial distance (in m) of the wheels relative to the center of the mobile robot, and $\left(\delta_{a},\delta_{b},\delta_{c}\right)$ are the angular orientation (in arc degrees) of each omnidirectional wheel, relative to the mobile robot reference frame $\left(X_{R},Y_{R}\right)$ .

The motion and trajectory of an omnidirectional mobile robot is defined by a basic motion vector,  M=(v,α,ω) , where v is the translational velocity of the displacement (in m/s), α is the angular orientation of the displacement (in arc degrees) relative to the robot reference frame $\left(X_{R},Y_{R}\right)$ , and ω is the angular rotational speed (in rad/s) applied to the center of the omnidirectional mobile robot. This basic motion vector is implemented in the APR mobile robots as a motion command using $M=\left(v,\alpha ,\omega ,t_{r}\right)$ or $M=\left(v,\alpha ,\omega ,d_{r}\right)$ , where $t_{r}$ is the relative duration of the displacement (in s), and $d_{r}$ is the relative distance of the displacement (in m), with $v=d_{r}/t_{r}$ .

Figure 2b represents graphically the motion vector M=(v,α,ω) that originates the translation and rotation of the mobile robot reference frame $\left(X_{R},Y_{R}\right)$ . The translational velocity v , the angular orientation of the mobile robot α and the angular rotational speed of the center of the mobile robot ω are linked to the angular rotational speed of the wheels $\left(\omega_{a},\omega_{b},\omega_{c}\right)$ (in rad/s), the radius of each wheel $\left(r_{a},r_{b},r_{c}\right)$ (in m), the computed translational velocities of the wheels $\left(V_{a},V_{b},V_{c}\right)$ (in m/s), the radial distance from the wheels to the center of rotation of the mobile robot $\left(R_{a},R_{b},R_{c}\right)$ and the relative orientation of the wheels $\left(\delta_{a},\delta_{b},\delta_{c}\right)$ . The conventional assumption is that the triplet of the structural parameters of the mobile robot does not change during its normal operation: $r_{a}=r_{b}=r_{c}$ = 148 mm, $R_{a}=R_{b}=R_{c}$ = 195 mm, $\delta_{a}$ = 60°, $\delta_{b}$ = 180°, and $\delta_{c}$ = 300°. Then, the implementation of one specific motion $\left(v_{k}>0,\alpha_{k},\omega_{k}\right)$ requires a unique combination of angular rotational speeds of the three wheels $\left(\omega_{ak},\omega_{bk},\omega_{ck}\right)$ , and one specific combination of angular rotational speeds of the three wheels $\left(\omega_{ak},\omega_{bk},\omega_{ck}\right)$ generates a unique motion in the mobile robot $\left(v_{k},\alpha_{k},\omega_{k}\right)$ .

### 3.1. Motion Originated by the Execution of a Single Motion Command $M=\left(v,\alpha ,\omega ,t_{r}\right)$

**Figure 3:**
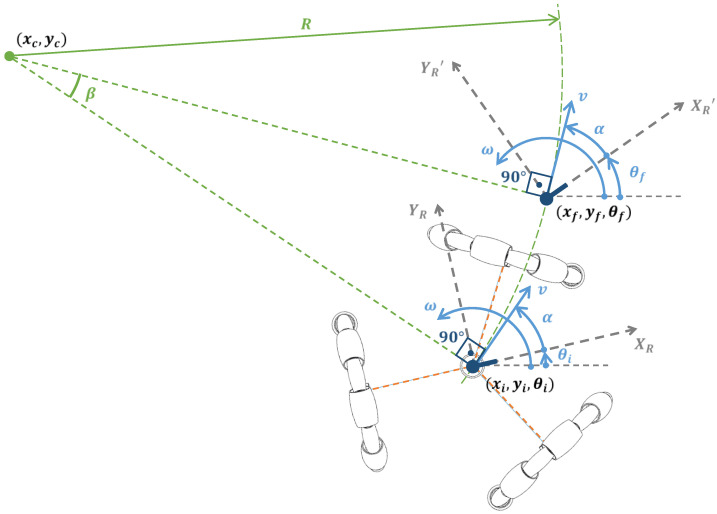
Representation of the displacement of an omnidirectional mobile robot located at $\left(x_{i},y_{i},\theta_{i}\right)$ when executing a motion command $\;M=\left(v,\alpha ,\omega ,t_{r}\right)$ . R is the radius of the circular trajectory, $\left(x_{c},y_{c}\right)$ is the location of the center of the circular trajectory, β is the angular displacement of the robot along the circular path, and $\left(x_{f},y_{f},\theta_{f}\right)$ is the final position of the robot.

Figure 3 is a representation of the kinematics of an ideal omnidirectional mobile robot with known initial position and orientation $\left(x_{i},y_{i},\theta_{i}\right)$ and a known single motion command $M=\left(v,\alpha ,\omega ,t_{r}\right)$ applied to the mobile robot. As a consequence of executing this motion command, an ideal omnidirectional mobile robot will move for a time, $t_{r}$ , with a translational velocity, v , starting the displacement in the relative angular direction defined by $\alpha +\theta_{i}$ , and with the center of the mobile robot rotating depending on the angular rotational velocity, ω , defined.

The final position of the mobile robot $\left(x_{f},y_{f},\theta_{f}\right)$ in the world frame after implementing a motion command $M=\left(v,\alpha ,\omega ,t_{r}\right)$ during a time $t_{r}$ is deterministic. The distance traveled with this motion command is:

(1) $$d=v\cdot t_{r}$$

**Figure 4:**
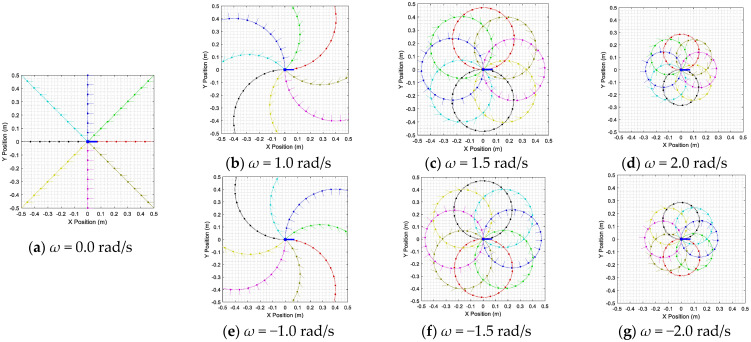
Simulation of the trajectories generated by motion commands $\;M=\left(v,\alpha ,\omega ,t_{r}=10s\right)$ . The starting point is (x=0,y=0,θ=0) , and the velocity v = 0.3 m/s. Showing 8 different angular orientations α : 0° (red), 45° (green), 90° (blue), 135° (cyan), 180° (black), 225° (yellow), 270° (magenta), 315° (olive), and different angular rotational speeds: (**a**) ω = 0.0 rad/s, (**b**) ω = 1.0 rad/s, (**c**) ω = 1.5 rad/s, (**d**) ω = 2.0 rad/s, (**e**) ω *=* −1.0 rad/s, (**f**) ω *=* −1.5 rad/s, (**g**) ω *=* −2.0 rad/s.

In the case of  ω≠0 , during the time $t_{r}$ required to execute the motion command $\;M=\left(v,\alpha ,\omega ,t_{r}\right)$ , the displacement of an omnidirectional mobile robot describes always a circular path (see Figure 4) whose radius  R is computed using:

(2) $$R=\frac{v}{\left|\omega \right|}$$

The location of the center of this circular path (valid during the time $\;t_{r}$ ) is computed using:

(3) $$x_{c}=-\frac{v}{\omega }\cdot \sin\left(\alpha +\theta_{i}\right)+x_{i}$$

(4) $$y_{c}=\frac{v}{\omega }\cdot \cos\left(\alpha +\theta_{i}\right)+y_{i}$$

The localization of the center of the circular path depends on the counterclockwise (ω>0) or clockwise (ω<0) direction of the angular rotational speed applied to the center of the mobile robot.

The final position of the mobile robot in the world frame $\left(x_{f},y_{f},\theta_{f}\right)$ is computed based on the angular displacement β along the circular path using:

(5) $$\beta =\left(\omega \cdot t_{r}\right)\omega \frac{360{}^{\circ}}{2\pi }$$

(6) $$x_{f}=\frac{v}{\omega }\cdot \sin\left(\theta_{i}+\alpha +\beta \right)+x_{c}$$

(7) $$y_{f}=-\frac{v}{\omega }\cdot \cos\left(\theta_{i}+\alpha +\beta \right)+y_{c}$$

(8) $$\theta_{f}=\theta_{i}+\omega \cdot t_{r}\cdot \frac{360{}^{\circ}}{2\pi }$$

Alternatively, in the case of ω=0 (no angular rotational speed), the radius,  R , of the circular path becomes infinite and the mobile robot moves only straight (see Figure 4). Then, the final position of the mobile robot in the world frame $\left(x_{f},y_{f},\theta_{f}\right)$ can be computed using:

(9) $$x_{f}=x_{i}+v\cdot t_{r}\cdot \cos\left(\theta_{i}+\alpha \right)$$

(10) $$y_{f}=y_{i}+v\cdot t_{r}\cdot \sin\left(\theta_{i}+\alpha \right)$$

(11) $$\theta_{f}=\theta_{i}$$

Figure 4 shows a simulation of the trajectories of an ideal omnidirectional mobile robot executing one specific motion command $M=\left(v,\alpha ,\omega ,t_{r}\right)$ during a long execution time: $\;t_{r}=10\;s$ . Each trajectory of the mobile robot is represented with a thin line while the bold point and bold line are sparse representations of the position and orientation of the mobile robot during the displacement. Each particular trajectory is depicted with an identifying color. In all the motion commands simulated, the starting position of the mobile robot is the same $\left(x_{i}=0,y_{i}=0,\theta_{i}=0\right)$ , and the duration of the displacement $\;t_{r}$ and the translational velocity  v of the motion are also the same in all cases. The motion commands represented in each plot have angular orientations α from 0 to 315° in increments of 45° (labeled with the same identifying color). Each plot represents a different angular rotational speed ω from 0 to 2.0 rad/s and from 0 to −2.0 rad/s. In Figure 4a, case  ω=0 , the mobile robot performs straight displacements in the direction established by the value of the angular orientation  α . The other cases are for ω≠0 , and then the mobile robot describes a circular trajectory defined by the value of the radius  R , which is lower as ω increases. A common characteristic of the basic trajectories shown in Figure 4 is that the orientation of the mobile robot relative to the tangent of the trajectory is constant during the motion, a typical feature of omnidirectional mobile robots.

### 3.2. Estimation of the Motion Command to Reach a Target Position $\left(x_{f},y_{f},\theta_{f}\right)$ for a Known v

**Figure 5:**
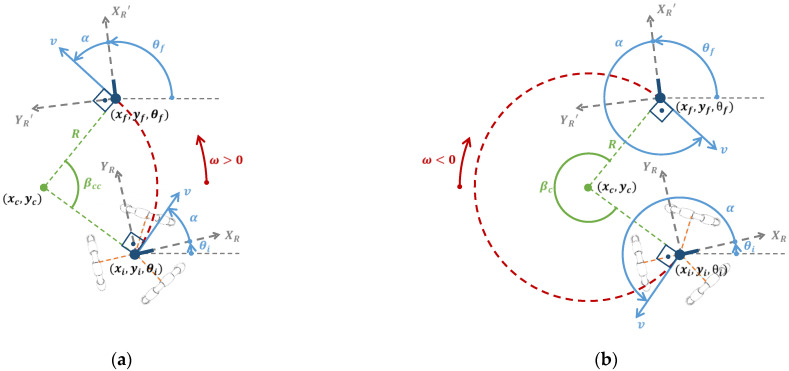
Representation of the trajectory of the mobile robot: (**a**) case with translation and rotation in the counterclockwise direction (ω>0) and (**b**) case with translation and rotation in the clockwise direction (ω<0) .

Alternatively to the previous section, Figure 5 is a representation of the kinematics of an omnidirectional mobile robot with a known initial position $\left(x_{i},y_{i},\theta_{i}\right)$ , known final destination $\left(x_{f},y_{f},\theta_{f}\right)$ and known target translational velocity v of the motion. The unknown parameters of the motion command $M=\left(v,\alpha ,\omega ,t_{r}\right)$ required to reach the final destination are α,ω and $t_{r}$ , and the determination of these parameters is also deterministic.

There are four possible case trajectories depending on the value of the target angular rotational speed ω : (1) translation and rotation in the counterclockwise direction (ω>0) (Figure 5a), (2) translation and rotation in the clockwise direction (ω<0) (Figure 5b), (3) translation without rotation (v≠0,ω=0) and (4) rotation without translation (v=0,ω≠0) .

#### 3.2.1. Translation and Rotation in the Counterclockwise Direction (ω>0) for a Known v

Figure 5a depicts the trajectory of an ideal omnidirectional mobile robot (red dotted line) with a known initial position $\left(x_{i},y_{i},\theta_{i}\right)$ , known final position, known translational velocity v and a target counterclockwise trajectory condition (ω>0) . The unknown parameters of the motion are the values of α,ω and $t_{r}$ . The counterclockwise condition (ω>0) means that the angular orientation of the mobile robot will increase from $\theta_{i}$ to $\theta_{f}$ . In the case of $\theta_{i}>\theta_{f}$ , the trajectory must go from $\theta_{i}$ to $\theta_{f}+360{}^{\circ}$ .

In this case, the angle covered by the circular trajectory $\beta_{cc}$ and the radius R of the circular trajectory are computed as:

(12) $$if\;(\theta_{f}>\theta_{i})\;then\;\beta_{cc}=\theta_{f}-\theta_{i}\;if\;\left(\;\theta_{f}<\theta_{i}\;\right)\;then\;\beta_{cc}=\left(\theta_{f}-\theta_{i}\right)+360{}^{\circ}$$

(13) $$R=\sqrt[{2}]{\frac{{\left(X_{f}-X_{i}\right)}^{2}+{\left(Y_{f}-Y_{i}\right)}^{2}}{2\cdot \left(1-cos\left(\beta_{cc}\right)\right)}}$$

The angular velocity required to complete this counterclockwise motion is:

(14) $$\omega =\frac{v}{R}$$

The angular orientation of the velocity vector required is:

(15) $$\alpha =\frac{180-\beta_{cc}}{2}+\mathrm{atan}\left(\frac{Y_{f}-Y_{i}}{X_{f}-X_{i}}\right)-\theta_{i}-90{}^{\circ}$$

Finally, the exact time required to complete this displacement is:

(16) $$tr=\frac{\beta_{cc}}{\omega }$$

#### 3.2.2. Translation and Rotation in the Clockwise Direction (ω<0) for a Known v

Alternatively to the previous case, Figure 5b depicts the trajectory of an ideal omnidirectional mobile robot (red dotted line) with a known initial position $\left(x_{i},y_{i},\theta_{i}\right)$ , known final destination $\left(x_{f},y_{f},\theta_{f}\right)$ , known target translational velocity v and a target clockwise trajectory condition (ω<0) . Then the unknown parameters of the motion are the values of α,ω and $t_{r}$ .

In this case, the angle covered by the circular trajectory $\beta_{c}$ and the radius R of the circular trajectory are computed as:

(17) $$if\;\left(\;\theta_{f}>\theta_{i}\;\right)\;then\;\beta_{c}=\left(\theta_{f}-\theta_{i}\right)-360{}^{\circ}\;if\;(\theta_{f}<\theta_{i})\;then\;\beta_{c}=\theta_{f}-\theta_{i}$$

(18) $$R=\sqrt[{2}]{\frac{{\left(X_{f}-X_{i}\right)}^{2}+{\left(Y_{f}-Y_{i}\right)}^{2}}{2\cdot \left(1-cos\left(\beta_{c}\right)\right)}}$$

The angular velocity required to complete this clockwise motion is:

(19) $$\omega =-\frac{v}{R}$$

The angular orientation of the velocity vector required is:

(20) $$\alpha =-\left(\frac{180+\beta_{c}}{2}\right)-\mathrm{atan}\left(\frac{X_{i}-X_{f}}{Y_{i}-Y_{f}}\right)-\theta_{i}$$

Finally, the time required to complete this displacement is again:

(21) $$tr=\frac{\beta_{c}}{\omega }$$

#### 3.2.3. Translation without Rotation (v≠0,ω=0) for a Known v

When the initial and final angular orientations of the omnidirectional mobile robot are the same $\left(\theta_{i}=\theta_{f}\right)$ , the angular rotational speed of the motion command is zero (ω=0) , and then the trajectory of the mobile robot defines a straight line. The unknown parameters of the motion command are the values of α and $t_{r}$ . In this special case, the distance traveled d during this translation is computed with:

(22) $$d=\sqrt[{2}]{{\left(X_{f}-X_{i}\right)}^{2}+{\left(Y_{f}-Y_{i}\right)}^{2}}$$

and the angular orientation of the velocity α required to complete this motion is:

(23) $$\alpha =\mathrm{atan}\left(\frac{Y_{f}-Y_{i}}{X_{f}-X_{i}}\right)-\theta_{i}$$

Then, the time required to complete this translation is:

(24) $$tr=\frac{d}{v}$$

#### 3.2.4. Static Rotation without Translation (v=0,ω≠0)

The last alternative is a special case that defines a simple static rotation of the mobile robot without any additional translation, and thus the linear translational velocity v must be zero. In this static rotation, the angular orientation of the translational speed α has no effect on the motion; therefore its value is indifferent. This static rotation depends on the angular rotational speed ω and the target final angular orientation of the mobile robot $\theta_{f}$ ; thus these parameters must be defined, and then the unknown parameter of the motion is only the value of $t_{r}$ . In this static case, the increment of the angular position ( β ) can be calculated as in Section 3.2.3 and Section 3.2.4: according to the direction of rotation (counterclockwise, (ω>0) , or clockwise, (ω<0) ) and depending on the initial and final angular positions $(\theta_{i}>\theta_{f}$ or $\theta_{i}<\theta_{f})$ .

Nevertheless, in practice, this special case can be highly simplified by defining the angular rotational speed of the rotation ω and an additional parameter that is the relative increment of the angular orientation of the mobile robot β . Then, the sign of the angular rotation β directly defines the direction of rotation (instead of the sign of ω ). Therefore, if β>0 , the robot must rotate in the counterclockwise direction (requiring ω>0 ), and if β<0 , the robot must rotate in the clockwise direction (requiring ω<0 ). Finally, the time required to complete the rotation based on this definition is:

(25) $$tr=\frac{\left|\beta \right|}{\left|\omega \right|\cdot \frac{360{}^{\circ}}{2\pi }}$$

The angular rotational speed of the angular rotation ω applied internally by the control system of the mobile robot must be:

(26) ω=sign(β)·|ω|

### 3.3. Kinematic Model: Determination of $\left(\omega_{a},\omega_{b},\omega_{c}\right)$ from (v,α,ω)

The analysis of the kinematic model of the omnidirectional motion system allows the determination of the angular rotational speeds of the three omnidirectional wheels $\left(\omega_{a},\omega_{b},\omega_{c}\right)$ required to implement a specific motion command M=(v,α,ω) . The kinematic model of the motion system of the mobile robot APR was described in Moreno et al. [18] and can also be summarized as follows [9] (see Figure 3).

(27) $$v_{x}=v\cdot \cos\left(\alpha \right)$$

(28) $$v_{y}=v\cdot \sin\left(\alpha \right)$$

(29) $$IK=\left[\begin{array}{ccc} -\sin\left(\delta_{a}\right) & \cos\;\left(\delta_{a}\right) & R_{a}\\ -\sin\left(\delta_{b}\right) & \cos\;\left(\delta_{b}\right) & R_{b}\\ -\sin\left(\delta_{c}\right) & \cos\;\left(\delta_{c}\right) & R_{c} \end{array}\right]$$

(30) $$\left[\begin{array}{c} V_{a}\\ V_{b}\\ V_{c} \end{array}\right]=IK\cdot {\left[\begin{array}{c} v_{x}\\ v_{y}\\ \omega \end{array}\right]}_{Robot}$$

(31) $$\left[\begin{array}{c} \omega_{a}\\ \omega_{b}\\ \omega_{c} \end{array}\right]=\left[\begin{array}{ccc} \frac{1}{r_{a}} & 0 & 0\\ 0 & \frac{1}{r_{b}} & 0\\ 0 & 0 & \frac{1}{r_{c}} \end{array}\right]\left[\begin{array}{c} V_{a}\\ V_{b}\\ V_{c} \end{array}\right]$$

where $\left(V_{a},V_{b},V_{c}\right)$ are the linear speed of each wheel (in m/s) and $\left(\omega_{a},\omega_{b},\omega_{c}\right)$ the angular velocities of each wheel (in rad/s). The brushed DC motors that drive the wheels of the mobile robot have a gear ratio of 64:1, and the internal PID controllers of the motion control board use targets defined in rpm. Therefore, the target angular rotational speeds of the motors $\left(\omega_{MA},\omega_{MB},\omega_{MC}\right)$ (defined in rpm) are:

(32) $$\left[\begin{array}{c} \omega_{MA}\\ \omega_{MB}\\ \omega_{MC} \end{array}\right]=\left[\begin{array}{c} \omega_{a}\\ \omega_{b}\\ \omega_{c} \end{array}\right]\cdot \frac{60}{2\pi }\cdot \frac{64}{1}$$

### 3.4. Kinematic Model: Determination of (v,α,ω) from $\left(\omega_{a},\omega_{b},\omega_{c}\right)$

The analysis of the kinematic model of the omnidirectional motion system allows the determination of the motion (v,α,ω) based on the angular rotational speed of the three omnidirectional wheels $\left(\omega_{a},\omega_{b},\omega_{c}\right)$ . The computation of this kinematic model was described in Moreno et al. [18]. The angular rotational speed of the wheels is deduced from the information gathered by the rotary encoders attached to the geared brushed DC motors. Therefore, the estimate of the angular rotational speed of the motors $\left(\omega_{MA},\omega_{MB},\omega_{MC}\right)$ can be converted to the angular rotational speed of the wheels $\left(\omega_{a},\omega_{b},\omega_{c}\right)$ using:

(33) $$\left[\begin{array}{c} \omega_{a}\\ \omega_{b}\\ \omega_{c} \end{array}\right]=\left[\begin{array}{c} \omega_{MA}\\ \omega_{MB}\\ \omega_{MC} \end{array}\right]\cdot \frac{2\pi }{60}\cdot \frac{1}{64}$$

Then, the translational velocity of the center of the mobile robot referred to the mobile robot reference frame $\left(v_{x},v_{y}\right)$ and its angular rotational speed ω can be computed by inverting the inverse kinematic matrix of the mobile robot. In the case of omnidirectional wheels with the same radius r :

(34) $${\left[\begin{array}{c} v_{x}\\ v_{y}\\ \omega \end{array}\right]}_{Robot}=IK^{-1}\cdot \left[\begin{array}{c} \omega_{a}\\ \omega_{b}\\ \omega_{c} \end{array}\right]\cdot r$$

where the linear translational velocity, v , of the mobile robot and the angular orientation of the translational velocity, α , referred to the mobile reference frame are computed using:

(35) $$v=\sqrt{v_{x}^{2}+v_{y}^{2}}$$

(36) $$\alpha =\tan^{-1}\left(\frac{v_{y}}{v_{x}}\right)$$

Finally, in an ideal implementation of the omnidirectional mobile robot structure with an exact mechanical placement of the wheels ( $R=R_{a}=R_{b}=R_{c}$ and $\delta_{a}$ = 60°, $\delta_{b}$ = 180°, $\delta_{c}$ = 300°) this matrix $IK^{-1}$ is:

(37) $$IK^{-1}=\left[\begin{array}{ccc} -\frac{1}{\sqrt{3}} & 0 & \frac{1}{\sqrt{3}}\\ \frac{1}{3} & -\frac{2}{3} & \frac{1}{3}\\ \frac{1}{3R} & \frac{1}{3R} & \frac{1}{3R} \end{array}\right]$$

### 3.5. Odometry: Determination of (Δx,Δy,Δθ) from $\left(\omega_{a},\omega_{b},\omega_{c}\right)$

Odometry is the use of the information of the rotation of the wheels of a mobile robot to estimate their position relative to a starting location. This method is very effective, but it is also very sensitive to errors because it is based on the discrete integration of the information of the velocity of the wheels [26,27]. The odometry used in the omnidirectional mobile APR-02 is based on the kinematic analysis described in the previous Section 3.4. This kinematic model converts the information of the angular rotational speed of the wheels of the omnidirectional mobile robot $\left(\omega_{a},\omega_{b},\omega_{c}\right)$ into an estimation of the motion of the mobile robot $\left(v_{x},v_{y},\omega \right)$ relative to the robot reference frame. Then, this motion can be combined with the previous known position of the mobile robot $\left(x_{i},y_{i},\theta_{i}\right)$ in order to update the current location of the mobile robot.

Typically, the odometry of a mobile robot provides a new update of the estimation of the position of the robot (x,y,θ) in the world reference frame after a fixed time lapse ΔT . This new position of the robot can be computed using the expressions provided in Section 3.1 or directly applying a compact transformation matrix. In this second case, the velocity of the center of the robot $\left(v_{x},v_{y}\right)$ relative to the mobile robot reference frame is converted into the world reference frame according to the previous angular orientation of the mobile robot, $\theta_{i}$ :

(38) $${\left[\begin{array}{c} v_{X}\\ v_{Y}\\ \omega \end{array}\right]}_{World}=\left[\begin{array}{ccc} \cos\left(\theta_{i}\right) & -\sin\left(\theta_{i}\right) & 0\\ \sin\left(\theta_{i}\right) & \cos\left(\theta_{i}\right) & 0\\ 0 & 0 & 1 \end{array}\right]\cdot {\left[\begin{array}{c} v_{x}\\ v_{y}\\ \omega \end{array}\right]}_{Robot}$$

Since the next position of the robot is calculated after a time lapse ΔT *,* this new estimate of $\left(v_{x},v_{y},\omega \right)$ is an average value computed during the time lapse ΔT . Then, the general assumption is that ΔT is small enough and the information provided by the rotary encoders is precise enough in order to consider the new value of $\left(v_{x},v_{y},\omega \right)$ as representative of the motion of the mobile robot. Finally, the displacement and new position of the mobile robot relative to the world reference frame is computed using:

(39) $${\left[\begin{array}{c} \Delta x\\ \Delta y\\ \Delta \theta \end{array}\right]}_{World}={\left[\begin{array}{c} v_{X}\\ v_{Y}\\ \omega \end{array}\right]}_{World}\cdot \Delta T$$

(40) $${\left[\begin{array}{c} x_{f}\\ y_{f}\\ \theta_{f} \end{array}\right]}_{World}={\left[\begin{array}{c} x_{i}\\ y_{i}\\ \theta_{i} \end{array}\right]}_{World}+{\left[\begin{array}{c} \Delta x\\ \Delta y\\ \Delta \theta \end{array}\right]}_{World}$$

## 4. Path Planning and Path Following

### 4.1. Rough Trajectory Definition through Waypoints

The definition of the trajectory of the mobile robot APR from a starting point $\left(x_{i},y_{i},\theta_{i}\right)$ to an ending point $\left(x_{f},y_{f},\theta_{f}\right)$ is based on the definition of one or several intermediate trajectory waypoints $\left(x_{w},y_{w}\right)$ . These waypoints can be manually generated over a map of the application scenario [19], for example, by direct indication of the destination $\left(x_{f},y_{f}\right)$ , by direct indication of some intermediate destinations $\left(x_{w},y_{w}\right)$ or indicating the intermediate destination and the desired mobile robot orientation $\left(x_{w},y_{w},\theta_{w}\right)$ . In the cases of having only a final destination $\left(x_{f},y_{f}\right)$ , the sequence of intermediate destinations $\left(x_{w},y_{w}\right)$ can be automatically obtained by using the A* (A-star) algorithm [19,28] or by the application of an artificial potential field algorithm [16].

### 4.2. Path Planning: Linearizing and Smoothing the Trajectory

The path-planning procedure used in the mobile robot APR-02 consists of linearizing and smoothing the trajectory defined by the waypoints $\;\left(x_{w},y_{w}\right)\;$ with the application of splines using a constant distance interval [29,30,31,32]. The result of this spline interpolation is a fine sequence of intermediate trajectory points $\left(x_{k},y_{k},\theta_{k}\right)$ that the robot must follow in order to move from the starting point $\left(x_{i},y_{i},\theta_{i}\right)$ to the final destination $\left(x_{f},y_{f},\theta_{f}\right)$ :

(41) $$\left(x_{i},y_{i},\theta_{i}\right)\to \;\ldots \to \left(x_{k},y_{k},\theta_{k}\right)\to \;\left(x_{k+1},y_{k+1},\theta_{k+1}\right)\to \;\ldots \to \left(x_{f},y_{f},\theta_{f}\right)$$

The motion capabilities of an omnidirectional motion system allow an uncorrelated or independent definition of the intermediate trajectory positions $\left(x_{k},y_{k}\right)$ and the mobile robot orientations $\left(\theta_{k}\right)$ :

(42) $$\left(x_{i},y_{i}\right)\to \;\ldots \to \left(x_{k},y_{k}\right)\to \;\left(x_{k+1},y_{k+1}\right)\to \;\ldots \to \left(x_{f},y_{f}\right)\;\left(\theta_{i}\right)\to \;\ldots \;\to \left(\theta_{k}\right)\;\;\;\;\;\;\to \left(\theta_{k+1}\right)\;\;\;\;\;\;\;\;\;\;\;\to \;\ldots \to \;\left(\theta_{f}\right)$$

Therefore, an omnidirectional mobile robot is able to keep the same orientation during the whole displacement $\left(\theta_{f}=\theta_{i}\right)$ , reach a specific final angular orientation $\left(\theta_{f}=90{}^{\circ}\right)$ , rotate the mobile robot during the displacement $\left(\theta_{f}=\theta_{i}+N\cdot 360{}^{\circ}\right)$ or maintain an orientation tangent to the planned trajectory $\left(\theta_{K}=\tan\left(\left(y_{k+1}-y_{k}\right)/\left(x_{k+1}-x_{k}\right)\right)\right)\;$ in order to define a humanlike smooth motion that is expected to be more socially accepted [33].

This linearizing and smoothing strategy is based on the assumption that the motion command required to move the omnidirectional mobile robot between two positions $\left(x_{k},y_{k},\theta_{k}\right)\to \left(x_{k+1},y_{k+1},\theta_{k+1}\right)$ with a known target translational velocity v can be analytically obtained using the procedures described in Section 3.2.

**Figure 6:**
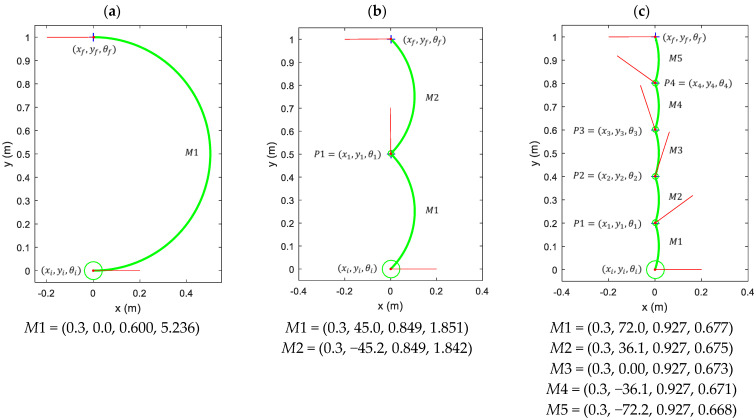
Representation of the motion command $M=\left(v,\alpha ,\omega ,t_{r}\right)$ and the mobile robot trajectory (green line) required to move from a starting point $P_{i}$ (green circle) to a planned destination $P_{f}$ depending on the number of intermediate waypoints defined using an interpolation procedure: (**a**) direct trajectory with only one motion command and no intermediate waypoints; (**b**) trajectory with one intermediate waypoint *P*1 that requires the computation of two motion commands; (**c**) trajectory with four intermediate waypoints *P*1, *P*2, *P*3 and *P*4 that require the computation of five motion commands.

Figure 6 illustrates the application of this path-planning procedure. Figure 6 shows an omnidirectional mobile robot that has to move from a starting point $P_{i}=\left(x_{i},y_{i},\theta_{i}\right)$ located at $\left(x_{i}=0,y_{i}=0,\theta_{i}=0\right)$ to a final destination point located at $\left(x_{f}=0,y_{f}=1,\theta_{f}=180{}^{\circ}\right)$ with an expected transversal linear velocity v fixed to 0.3 m/s. Figure 6a shows the implementation of this displacement using only a single motion command *M*1 (computed as defined in Section 3.2). In this example, the angular orientation of the mobile robot changes 180° $\left(\theta_{i}=0{}^{\circ},\;\theta_{f}=180{}^{\circ}\right)$ , and thus this single motion *M*1 will require a certain angular velocity (ω≠0) to rotate the mobile robot that will generate a curved trajectory displacement (see Figure 6a). In this case, this single motion *M*1 needs an execution time $t_{r}$ of 5.236 s in order to reach the destination. Alternatively, Figure 6b shows the same displacement using one intermediate trajectory point *P*1 interpolated in the middle of the displacement. In this case, the first motion command *M*1 is required to reach the intermediate trajectory point, and then the second motion command *M*2 is required to reach the destination. This trajectory is then composed of two consecutive circular displacements, but the inclusion of the intermediate point *P*1 has reduced significantly the arc of the circular trajectory of the mobile robot. Finally, Figure 6c shows the effect of using four interpolated trajectory points *P*1, *P*2, *P*3 and *P*4 in a planned direct displacement from $P_{i}$ to $P_{f}$ . In this case, the arc of the trajectory is almost inexistent, and the execution of this displacement is visually perceived as a single straight displacement in which the mobile robot is rotating. Therefore, increasing the number of intermediate interpolated trajectory points between $P_{i}$ and $P_{f}$ , the trajectory of the mobile robot becomes continuous without trajectory discontinuities. In the APR-02, the minimum value of the separation distance between intermediate interpolated trajectory points was obtained by trial and error as 55 mm. This value depends largely on the setting time of the PID that controls the velocity of the wheels. This setting time is 0.5 s in the case of the APR-02. The example case shown in Figure 6 defines a displacement between $P_{i}$ and $P_{f}$ , but the intermediate interpolated points can define a direct trajectory or any arbitrary trajectory.

### 4.3. Path-Following Procedure

The path-following procedure applied in the mobile robot APR-02 uses the current mobile robot position and orientation $\left(x_{p},y_{p},\theta_{p}\right)$ updated by the SLAM procedure and close to the current target position $\left(x_{k},y_{k},\theta_{k}\right)$ in order to compute the next motion command $M=\left(v,\alpha ,\omega ,t_{r}\right)$ required to reach the next planned intermediate trajectory point $\left(x_{k+1},y_{k+1},\theta_{k+1}\right)$ . This computation is repeated until reaching the final destination point. Therefore, the goal of this procedure is always to have an updated motion command in order to go to the next planned intermediate trajectory point instead of trying to pass precisely over an intermediate trajectory point.

The implementation of this path-following procedure in the mobile robot APR-02 is based on the precise position feedback provided by the onboard LIDAR rather than on the use of the prediction horizon proposed in model predictive control (MPC) approaches (see [34] for a comprehensive review). The use of the information of the odometry as position feedback in this path-following procedure was also tested but it had to be discarded because of the well-known effect of the cumulated errors generated in the odometry during a large displacement.

Finally, the main advantage of the proposed path-following procedure is the automatic compensation of the motion errors caused by wheel slippage without requiring additional specific compensation procedures [13]. Another advantage of this path-following procedure is the ability to maintain a constant translational velocity v during the whole displacement, a feature that is expected to increase the social acceptance of a mobile robot operating in a shared space with humans [33].

## 5. Experimental Evaluation of the Path-Tracking Accuracy

This section empirically evaluates the path-tracking accuracy of the path-planning and path-following procedures implemented in the mobile robot APR-02. As cited previously, this paper is inspired by the work of Li et al. [13] that proposed the evaluation of the path-tracking performances of an omnidirectional mobile robot using multiple Mecanum wheels completing circular and eight-shaped target trajectories. This evaluation was a pending task in the case of the mobile robot APR-02. The specific trajectories selected by Li et al. [13] are especially interesting. In the case of performing a circular trajectory, an omnidirectional mobile robot is able to complete this path by using only one motion command M=(v,α,ω) (see Figure 4), and thus this trajectory represents an easy path to follow. Alternatively, in the case of performing an eight-shaped trajectory, an omnidirectional mobile robot must continuously update the motion command $M=\left(v,\alpha ,\omega ,t_{r}\right)$ , and thus this is a very challenging trajectory prone to control errors.

**Figure 7:**
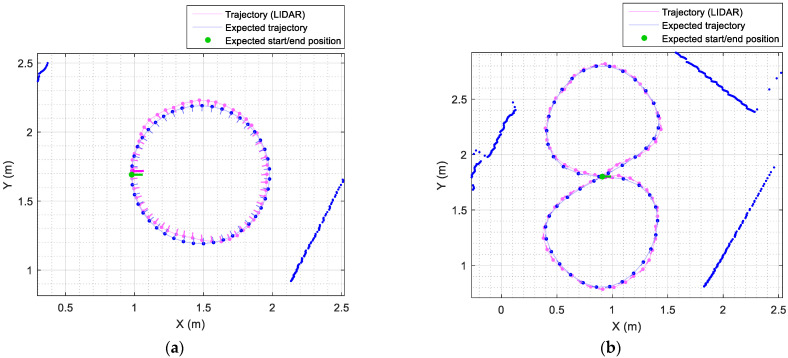
Comparison between the planned (blue line) and real (magenta) trajectories of the mobile robot moving at a constant speed of 0.15 m/s. The green line depicts the expected initial and final positions and orientations of the mobile robot: (**a**) describing a circular trajectory with the robot facing inward and (**b**) describing an eight-shaped trajectory with the robot facing forward.

**Figure 8:**
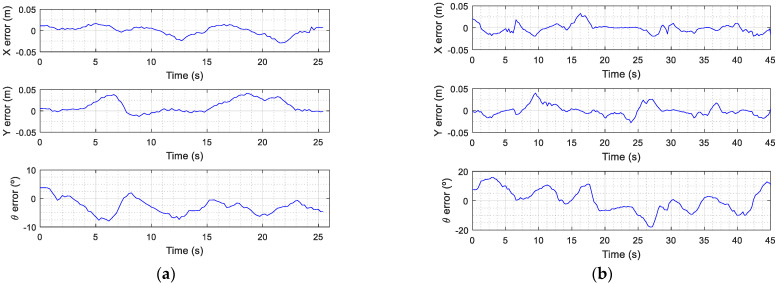
Error location $\left(x_{e},y_{e},\theta_{e}\right)$ when the mobile robot moves at a constant translational velocity of 0.15 m/s: (**a**) following a circular trajectory with the robot facing inward and (**b**) following an eight-shaped trajectory with the robot facing forward.

Figure 7 shows the circular and eight-shaped target trajectories used to evaluate the path-tracking accuracy of the omnidirectional mobile robot APR-02. Figure 7a shows a circular target trajectory (blue color) with a radius of 1 m. Figure 7a includes a sparse representation of the intermediate trajectory points $\left(x_{k},y_{k},\theta_{k}\right)$ (blue point and blue line) used to plan this trajectory. Figure 7a also shows the ground truth trajectory (magenta color) obtained when the mobile robot APR-02 completes this trajectory with a linear translational velocity v of 0.15 m/s, which is a very low velocity for a human-sized mobile robot. This ground truth trajectory was obtained from the information registered by the onboard LIDAR. Figure 7a also depicts a sparse representation of the real mobile robot position and orientation (magenta point and magenta line) that are correlated with the intermediate trajectory points planned. The detailed evolution of the location and orientation errors $\left(x_{e},y_{e},\theta_{e}\right)$ obtained in this experiment are also plotted in Figure 8a. Similarly, Figure 7b shows an eight-shaped target trajectory (blue color) with a distance between focus of 1 m. Figure 7b includes a sparse representation of the intermediate trajectory points $\left(x_{k},y_{k},\theta_{k}\right)$ (blue point and blue line) used to plan this trajectory. Figure 7b also shows the ground truth trajectory (magenta color) obtained when the mobile robot APR-02 completes this trajectory with a linear translational velocity v of 0.15 m/s. The detailed evolution of the location and orientation errors $\left(x_{e},y_{e},\theta_{e}\right)$ obtained in this experiment are plotted in Figure 8b.

Finally, Table 1 and Table 2 summarize the root mean square (RMS) error (RMSE) and maximum absolute error of the Euclidean distance between the expected and real mobile robot locations and between the expected and real mobile robot angular orientations. Table 1 shows the results obtained when performing a circular target trajectory and Table 2 the results obtained when performing an eight-shaped target trajectory. These two tables summarize the errors obtained with different translational velocities v where 0.10 m/s is visually perceived as a very slow velocity, 0.30 m/s is the nominal velocity that is visually perceived as normal or adequate for the mobile robot APR-02, and 0.5 m/s is externally visually perceived as a very fast (and maybe annoying) velocity. A video prepared by the authors showing the mobile robot APR-02 completing these two target trajectories at a translational velocity of 0.3 m/s is available on YouTube [35].

## 6. Discussion and Conclusions

This paper presents the empirical evaluation of the path-tracking accuracy of the omnidirectional mobile robot APR-02 designed to develop services as a personal assistant. This mobile robot uses three omnidirectional wheels driven by geared brushed DC motors with magnetic rotary encoders attached and is able to move in any direction while simultaneously changing its orientation. This paper describes the kinematics and path-planning procedure implemented in the mobile robot and empirically evaluates its path-tacking accuracy. The mobile robot uses a path-following procedure based on the self-location capabilities provided by a precise onboard LIDAR. The ground truth trajectory of the mobile robot during the experiments has been obtained by registering the information gathered by the onboard LIDAR. The experimentation area used to conduct the experiments included large plain walls in order to maximize the precision of the self-locating procedure [25].

Results of Table 1 show that the RMS error of the distance measured when performing a circular trajectory has its minimum value (0.017 m) for a translational velocity of 0.1 m/s, which is a very low velocity for a humanlike mobile robot. The RMS distance error has a slightly increasing tendency for translational velocities in the range from 0.25 to 0.5 m/s: between 0.033 m and 0.051 m. In this translational velocity range, the maximum distance error is in a range from 0.06 m to 0.12 m. This distance error can be interpreted as low for a mobile robot with a base diameter of 0.54 m and 31 kg. Results of Table 1 also show that the RMS of the angular orientation error of the mobile robot is always close to 6.5° and the absolute value of the maximum error is close to 13° in all the velocity ranges analyzed. We observed that this angular difference is due to the fact that the mobile robot always aligns its orientation with its trajectory, and thus a correction in the trajectory suddenly increases the angular error of the mobile robot. This current implementation produces smooth visual trajectories, but this effect will be analyzed in depth in future works in order to effectively reduce the angular error of the mobile robot. A video sequence showing the mobile robot APR-02 completing a circular trajectory while rotating is available in [35].

Results of Table 2 show that the RMS error in the distance measured when performing an eight-shaped trajectory monotony increases as the translational velocity increases. This trajectory is very challenging for any type of mobile robot because the speed of the wheels must be continuously adapted during this motion. The RMS distance error increased one order of magnitude in the velocity range from 0.1 to 0.5 m/s with values from 0.017 to 0.100 m. At low velocities, the absolute maximum error is around 0.44 m, at the nominal velocity of 0.3 m/s, the maximum error is 0.088 m, and the difficulty of this trajectory is evidenced at the velocity of 0.50 m/s with an instantaneous maximum distance error of 0.265 m. Again, the trajectory of the mobile robot is perceived as smooth, and the trajectory corrections are not visually detected. Results of Table 2 also show that the RMS of the angular orientation error of the mobile robot is around 9° or 10° with absolute maximum angular errors around 20° in all velocity ranges because this angular error is caused when correcting the trajectory of the mobile robot. A video sequence showing the mobile robot APR-02 completing an eight-shaped trajectory is available at [35].

The comparison of the path-tracking errors with the results of the scientific literature was complicated because of the different metrics used, the different sizes and weights of the mobile robots and the different motorization used. The comparative proposal of Li et al. [13] evaluated the path-following performances of omnidirectional mobile robots using four Mecanum wheels, evaluating the relative error of the displacement as a metric but without specifying the error in the angular orientation of the mobile robot. In [13], a circular trajectory with a radius of 1 m at a velocity of 0.2 m/s produced a relative error of the displacement of −15.23% in the X-axis and −2.85% in the Y-axis; this error was reduced to −1.99% in the X-axis and −1.50% in the Y-axis when applying a velocity compensation coefficient, a strategy that was not applied in this work. In [13], an eight-shaped trajectory at a velocity of 0.2 m/s produced relative errors of −13.91% and −1.17% and −1.95% and −0.04% when applying a tuned velocity compensation coefficient.

In general, the results obtained in this paper are in the same range as the results obtained by Li et al. [13] using a translational velocity of 0.2 m/s: absolute distance error of 0.050 m in the circular trajectory and 0.039 m in the eight-shaped trajectory. Then, the new contribution of this paper is the evaluation of the path-tracking accuracy at different translational velocities and the analysis of the error in the angular orientation of the mobile robot, which is fundamental information for an omnidirectional mobile robot. Another improvement relative to the proposal of Li et al. [13] is the avoidance of remote-control procedures applied to control the path of the mobile robot analyzed. However, a future pending work is the validation of the effect of tuned velocity compensation coefficients in path-tracking accuracy.

In this paper, the evaluation results of the path-tracking accuracy of the three-wheeled omnidirectional mobile robot APR-02 obtained when performing a circular trajectory at the nominal translational velocity of 0.3 m/s are: RMS distance error of 0.032 m, absolute distance error of 0.077 m, RMS angular orientation error of 6.27° and absolute angular orientation error of 12.60°. In the case of a more challenging eight-shaped trajectory, these values are: RMS distance error of 0.039 m, absolute distance error of 0.088 m, RMS angular orientation error of 7.76° and absolute angular orientation error of 21.51°. These small trajectory errors summarize the good visual impression generated by the displacement of the mobile robot APR-02.

Future research will be focused on the evaluation of the performance of the path-planning procedure of the mobile robot passing through open doors and maneuvering in a crowded environment. One of the goals will be the analysis of the affinity generated by the displacement of the mobile robot.

## Figures and Tables

**Table 1:** Path-tracking errors obtained in the case of performing a circular target trajectory (Figure 7a).

Speed (m/s)	Distance (m)		Angular Orientation (°)	
	RMSE	Absolute Maximum Error	RMSE	Absolute Maximum Error
0.10	0.017203	0.042762	6.7002	13.4761
0.15	0.021478	0.043392	3.9457	7.9026
0.20	0.023732	0.050270	5.6000	12.8638
0.25	0.033889	0.060157	5.3974	10.8083
0.30	0.032467	0.077929	6.2730	12.6040
0.35	0.051998	0.125830	7.3219	15.2344
0.40	0.038150	0.080402	6.3755	12.9896
0.45	0.040882	0.101140	6.0992	11.1705
0.50	0.032762	0.070848	8.0095	17.6987

**Table 2:** Path-tracking errors obtained in the case of performing an eight-shaped target trajectory (Figure 7b).

Speed (m/s)	Distance (m)		Angular Orientation (°)	
	RMSE	Absolute Maximum Error	RMSE	Absolute Maximum Error
0.10	0.017036	0.045863	8.99090	20.8563
0.15	0.015341	0.044073	7.80650	17.9776
0.20	0.017418	0.039401	7.08460	17.6608
0.25	0.025817	0.068124	6.53130	17.2021
0.30	0.039706	0.088557	7.76150	21.5102
0.35	0.059036	0.123140	7.64930	19.2291
0.40	0.065989	0.151920	9.48780	20.7115
0.45	0.087276	0.220810	11.5129	24.3387
0.50	0.100260	0.265240	12.2232	22.5929
