# Correction: Palacín et al. Evaluation of the Path-Tracking Accuracy of a Three-Wheeled Omnidirectional Mobile Robot Designed as a Personal Assistant. *Sensors* 2021, *21*, 7216

**DOI:** 10.3390/s24206543

**Published:** 2024-10-10

**Authors:** Jordi Palacín, Elena Rubies, Eduard Clotet, David Martínez

**Affiliations:** Robotics Laboratory, Universitat de Lleida, Jaume II, 69, 25001 Lleida, Spain; helenarubies@gmail.com (E.R.); eduard.clotet@udl.cat (E.C.); david.martinez@udl.cat (D.M.)

After publication of the research paper [1], the authors wish to make the following corrections. In the original publication, there was a mistake in the typeset of Equations (31)–(33). 

In Equation (31), the order of the product of the matrixes was not correct. The correct Equation (31) is:

(31) $$\left[\begin{array}{c} \omega_{a}\\ \omega_{b}\\ \omega_{c} \end{array}\right]=\left[\begin{array}{ccc} \frac{1}{r_{a}} & 0 & 0\\ 0 & \frac{1}{r_{b}} & 0\\ 0 & 0 & \frac{1}{r_{c}} \end{array}\right]\left[\begin{array}{c} V_{a}\\ V_{b}\\ V_{c} \end{array}\right]$$

In Equation (32), there was a mistake in the conversion. The correct Equation (32) is:

(32) $$\left[\begin{array}{c} \omega_{MA}\\ \omega_{MB}\\ \omega_{MC} \end{array}\right]=\left[\begin{array}{c} \omega_{a}\\ \omega_{b}\\ \omega_{c} \end{array}\right]\cdot \frac{60}{2\pi }\cdot \frac{64}{1}$$

In Equation (33), there was a mistake in the conversion. The correct Equation (33) is:

(33) $$\left[\begin{array}{c} \omega_{a}\\ \omega_{b}\\ \omega_{c} \end{array}\right]=\left[\begin{array}{c} \omega_{MA}\\ \omega_{MB}\\ \omega_{MC} \end{array}\right]\cdot \frac{2\pi }{60}\cdot \frac{1}{64}$$

The authors state that the scientific results and conclusions are unaffected. This correction was approved by the Academic Editor. The original publication has also been updated. The authors apologize for any inconvenience caused to the readers.
